# Romanian Maize (*Zea mays*) Inbred Lines as a Source of Genetic Diversity in SE Europe, and Their Potential in Future Breeding Efforts

**DOI:** 10.1371/journal.pone.0085501

**Published:** 2013-12-31

**Authors:** Dana Șuteu, Ioan Băcilă, Voichița Haș, Ioan Haș, Mihai Miclăuș

**Affiliations:** 1 National Institute of Research and Development for Biological Sciences, Cluj-Napoca, Romania; 2 Agricultural Research and Development Station, Turda, Romania; 3 University of Agricultural Sciences and Veterinary Medicine, Cluj-Napoca, Romania

## Abstract

Maize has always been under constant human selection ever since it had been domesticated. Intensive breeding programs that resulted in the massive use of hybrids nowadays have started in the 60s. That brought significant yield increases but reduced the genetic diversity at the same time. Consequently, breeders and researchers alike turned their attention to national germplasm collections established decades ago in many countries, as they may hold allelic variations that could prove useful for future improvements. These collections are mainly composed of inbred lines originating from well-adapted local open pollinated varieties. However, there is an overall lack of data in the literature about the genetic diversity of maize in SE Europe, and its potential for future breeding efforts. There are no data, whatsoever, on the nutritional quality of the grain, primarily dictated by the zein proteins. We therefore sought to use the Romanian maize germplasm as an entry point in understanding the molecular make-up of maize in this part of Europe. By using 80 SSR markers, evenly spread throughout the genome, on 82 inbred lines from various parts of the country, we were able to decipher population structure and the existing relationships between those and the eight international standards used, including the reference sequenced genome B73. Corroborating molecular data with a standardized morphological, physiological, and biochemical characterization of all 90 inbred lines, this is the first comprehensive such study on the existing SE European maize germplasm. The inbred lines we present here are an important addition to the ever-shrinking gene pool that the breeding programs are faced-with, because of the allelic richness they hold. They may serve as parental lines in crosses that will lead to new hybrids, characterized by a high level of heterosis, nationwide and beyond, due to their existing relationship with the international germplasm.

## Introduction

Maize (*Zea mays* ssp. *mays*) is the most important crop of the world in terms of production, surpassing rice and wheat, according to the 2012 figures by FAOSTAT (875 million metric tons, compared to 718 million metric tons for rice, and 674 million metric tons for wheat). Domesticated about 9000 years ago in the Rio Balsas region of Mexico [1,2], maize has been under constant human selection ever since. Teosinte (*Zea mays* ssp. *parviglumis*) is the wild relative of maize, and it has greatly contributed to increasing the genetic diversity of the latter through repeated inter-crosses. Therefore this tremendous crop had the ability to rapidly extend and adapt to different environments of the American continent, from the tropical regions, to the high altitudes of the Andes. However, that is not true for the Eastern Hemisphere (especially Europe) where Christopher Columbus introduced maize only in the late 15^th^ century, bringing the first germplasm from West Indies. Regardless of the speed that characterized its spread across the continent, maize still kept a low genetic diversity in Europe, due to the limited germplasm available when first introduced. Over the years, repeated crosses have been made with other germplasm from the American continent. Races were created, fully adapted to specific regions of Europe, characterized by specific environmental factors, like soil and climate. In Romania alone, four landrace complexes were defined, subdivided into 17 races and six sub-races [3]. By the beginning of the 17^th^ century maize was already mentioned as an extended crop in Romania, reaching up to 50% of country’s cultivated surface in the late 19^th^ century [4-6]. Inbred lines were created by recurrent self-pollinations. They were later used, especially after World War II, in creating hybrids. All these transformations reduced genetic diversity and resulted in the establishment of maize collections in many countries, not just Romania, in their efforts to preserve local germplasm. Collections like these are a valuable source for breeding programs but in order to serve their purpose they first have to be rigorously characterized and classified. 

The first morphological descriptions of such collections have started in the 60s and included Romania (reviewed in 7) but it was not until the 80s that the first isoenzyme studies were carried out, followed by Restriction Fragment Length Polymorphism (RFLP) markers, a decade later. But Romanian races were not well represented in such studies, with only three analyzed by means of RFLP [8].

 Recent studies on European maize populations do take into account the morphological diversity, but mainly focus on the molecular component, as the basis for predicting heterosis (i.e., the term coined to describe the better qualities of a progeny when compared to its parents) in newly developed hybrids. For example, Hartings et al., 2008 [9], analyzed 54 Italian landraces both morphologically and molecularly (using AFLP – Amplified Fragment Length Polymorphism - markers) and concluded that the latter method gives a better resolution on the relationships existing between the populations. Flint-Garcia et al., 2009 [10], investigated 300 hybrid genotypes for 17 morphological traits and concluded that one could maximize heterosis by crossing two genetically distant lines that come from similar environmental conditions. They also confirmed that a large genetic distance existing between the parents, translates into a high amount of heterosis. In this context a number of research groups have started dissecting the genetic diversity of maize in Europe, and not only, taking advantage of modern molecular biology tools and technologies. The favorite molecular markers for analyzing genetic diversity have been, and still are, SSRs (Simple Sequence Repeat) and SNPs (Single Nucleotide Polymorphism). Van Inghelandt et al., 2010 [11], used both types of markers in an analysis of genetic diversity and population structure of elite maize germplasm from Europe and North America and found that SSR markers were superior to SNPs, but both offered consistent results in terms of germplasm organization into heterotic groups. A couple of other studies, on the introduction of temperate maize in Europe [12] and introgressions from modern hybrids to landraces [13] have used SSR markers on 275 populations and 296 genotypes, respectively, but the number of loci interrogated was low: 24 and 21, respectively. The first study only included three Romanian populations, whereas the second only focused on the genetic diversity present in Italy. Another very recent study on 285 inbred lines originating from various areas of the globe aimed at predicting complex heterotic traits in maize but did not include any germplasm from SE Europe, and focused almost exclusively on German lines, plus a few French and Swiss entries [14]. 

There is an overall lack of data in the literature about the genetic diversity of maize in SE Europe, and its potential for future breeding efforts. There are no data, whatsoever, on the nutritional quality of the grain, primarily dictated by the zein proteins - the main storage proteins in maize kernels. This is due to zeins’ disproportionate levels of the 20 essential amino acids [15]. Zeins are classified based on their structure in α-, β-, γ-, and δ-zeins, and can be further subdivided based on their relative molecular mass into 22- and 19-kDa α-zeins, 15-kDa β-zeins, 50-, 27- and 16-kDa γ-zeins, and 18- and 10-kDa δ-zeins [16]. They have a characteristic pattern on an SDS-PAGE gel, with six bands clearly visible. These correspond to the 27-, 22-, 19-, 16-, 15-, and 10-kDa zeins. The 50-kDa zein is a single gene copy and its protein produces a faint band on gel, whereas the 18-kDa band is masked by the large amount of 19-kDa zein protein. When trying to improve grain quality one has to lower the zein content and thus facilitate the accumulation of albumins and globulins, which have a more balanced amino acid ratio. Researchers and breeders alike have tried to take advantage of one of maize’s wild ancestors in trying to improve this crop, by crossing teosinte with elite inbred lines and selecting for higher lysine, methionine, and phenylalanine content in hybrids [17], as amino acids that are deficient in maize flour. Another way of achieving this is by taking advantage of the existing maize germplasm, with emphasis on the century-old inbred lines of Eastern Europe. Valuable traits of such inbred lines can be easily incorporated into breeding programs, without the need of tedious crosses to teosinte. 

We therefore sought to use the Romanian maize germplasm as an entry point in understanding the molecular make-up of maize in this part of Europe. By using 80 SSR markers, evenly spread throughout the genome, on 82 inbred lines from various parts of the country, we were able to decipher population structure and existing relationships among them, and compared to a set of eight international standards (including the reference sequenced genome B73 [18]). Morphological, physiological, and biochemical descriptors were used to characterize each of the 90 inbred lines, according to the internationally accepted protocol “Guidelines for the development of crop descriptor lists” [19]. Complementary to crop descriptors above, 100 inbred lines (the 90 that were genotyped plus an extra ten) were investigated in terms of protein content, and more specifically, the zeins, as the main storage proteins in the kernel, and the ones having an impact on the nutritional quality of the grain. All data generated (molecular, morpho-physiological, and biochemical) can be easily incorporated into other future studies on the maize germplasm of other countries, or used in informative comparative studies, due to the standard descriptors and protocols followed. Most importantly, the data presented here open the way to future crosses with inbred lines of different countries that would not only increase gene diversity but also generate superior hybrids across the continent and beyond.

## Materials and Methods

### Plant material and DNA extraction

 We genotyped 90 inbred lines with 80 SSR markers. Included within the group, we analyzed 47 inbred lines that originate from various local populations of Romania, eight represent international standards (including the reference sequenced genome B73 [18]), whereas the rest are representative inbred lines currently being used in breeding programs within the country. All of them have been self-crossed at least ten times. Detailed information can be found in Table S1. For each of the 90 inbred lines ten seedlings were grown on filter paper for nine days, and five of those were sampled and desiccated in tubes filled with silica gel for 2-3 weeks, changing the silica gel at least once. The 450 samples were later milled in 2 ml Eppendorf tubes using 3 mm tungsten beads. 15 mg per sample were used for DNA extraction, with imnuPREP Plant DNA Kit (Analytik Jena), following the manufacturer’s protocol. Equal volumes of five samples per inbred line were pooled to form the template solutions used in SSR genotyping. The DNA quality was examined on agarose gels and quantified using NanoDrop.

### Morpho-physiological and biochemical descriptors

 The comprehensive characterization of the 90 inbred lines is in accordance to the strategic set of descriptors and passport data described in “Guidelines for the development of crop descriptor lists” [19]. Plant morphology is described by: height, insertion height of the main ear, total number of leaves, and percentage of sterile plants (i.e., plants without an ear). Ear morphology is described by: length, kernel efficiency, weight of 1000 kernels, kernel type, and cob color. The physiological descriptors used were: sum of temperatures to flowering, sum of temperatures to maturity, plant resistance to *Ostrinia nubilalis*. Protein, fat, starch, and fiber content in the kernels were also measured (Table S1). At least six plants in each experimental plot were sib-pollinated by pollen from the same plot, to avoid xenia effects. Approximately five hand-pollinated ears per row were harvested, after physiological maturity and bulked for chemical analysis. In addition, 50 grains were collected from the middle of each plot and used to measure moisture concentration. A representative sample of 50 g for each plot was ground and the concentration of starch, protein, oil, fiber and ash in the flour was determined with a Dickey-John Instalab 600 near-infrared reflectance analyzer, after curve calibration.

### SSR genotyping

 Each inbred line was scored with a set of 80 fluorescently dyed (6-FAM) SSR primer pairs that were evenly spread on the ten maize chromosomes. Their ID and chromosomal position are listed in Table S2 together with information on repeat type, forward and reverse sequences, optimal annealing temperature, and size of observed products. They have been used before in similar studies [20] and are freely available from MaizeGDB (www.maizegdb.org), section “Probes/molecular markers – SSRs”. Each primer pair had to be optimized in terms of its annealing temperature (ranging from 55 to 64 °C), as poor amplification or unspecific bands were otherwise present. PCR program used: 93 °C for 1 min, 93 °C for 30 sec, primer specific annealing temperature for 30 sec, 72 °C for 1 min, 30x steps 2 - 4, and final elongation at 72 °C for 5 min. The PCR products were purified on a mix of Sephadex - Sephacryl (1:1) (GE Healthcare Bio-Sciences AB) and then diluted 50x. 1.5 μl from this dilution were added to a 10 μl mix of HiDi formamide and GeneScan 500 ROX standard (Applied Biosystems) and then subjected to capillary electrophoresis on an ABI PRISM 3130 Genetic Analyzer (Applied Biosystems). GeneMapper v.4.0 software was used for scoring the alleles. 

### Data analysis

The output generated by GeneMapper v.4.0 consists of 80 text files: one for each of the SSR markers used to interrogate the 90 inbred lines. Two synthetic tables (Tables S3 and S4) were put together in the specific formats required by PowerMarker v.3.25 [21] and Structure 2.3.4 [22-24] software, respectively. 

PowerMarker v.3.25 was used to calculate the total number of alleles present in the 90 inbred lines, gene diversity, heterozygosity and polymorphism information content (PIC) at each of the 80 loci. A frequency matrix was built for each of the alleles and then used to calculate both Neighbor Joining (NJ) and by Unweighted Pair Group Method with Arithmetic Mean (UPGMA) phylogenetic trees, based on the Shared Allele distance method, with 1000 replications. The two consensus trees were built using MEGA v.5 [25].

The population structure of the 90 inbred lines was inferred with Structure v.2.3.4 software. In order to infer λ (allele frequency parameter), we first assumed one sub-group (K=1) and created a batch of five runs, each having both the length of the burnin period and the number of MCMC reps after burnin set to 100,000 according to Inghelandt et al., 2010 [11]. The mean value of λ, from the five runs, was used in a second batch-run where admixture was chosen as ancestry model, and correlated allele frequencies among pops as frequency model. Burnin time and number of iterations were both set to 100,000 and ten replications were performed for each K, from one to 15. The best K was determined using the ad hoc criterion proposed in Evanno et al., 2005 [26]. All of the above parameters were used to build the population structure based on data collected from ten to 70 SSR markers, in increments of ten, and increments of one SSR marker starting from 70 and up to the final number of 80 markers. After inferring the best value of K=2, the burnin time and number of iterations were both increased to 1,000,000 in order to compute the final population structure, based on data from 80 SSRs. The inbred lines were thus split into two big clusters and an admixed population. The latter contains inbred lines that had less than 90% calculated probability of being part of any of the two main clusters. Once the two clusters were defined, the same algorithm was used to infer the subgroups within.

For other statistical analyses (normality tests, t-test for normally distributed data, Mann-Whitney test for non-normal distribution) PAST v.2.17c 2013 software [27] was used. 

### Zein protein extraction

 Ten kernels from each of the 90 inbred lines were pooled and milled using a coffee grinder, then stored in 2 ml tubes. 100 mg of endosperm powder were mixed with 400 μl extraction buffer (70% ethanol, 2% β - mercaptoethanol) and kept at room temperature for 2 hours. After centrifuging at 13,000 rpm for 10 min, 200 μl of supernatant were transferred into a new tube. 10 μl of 10% SDS was added and then vacuum-dried for 45 min in a centrifuge. The samples were resuspended in 100 μl water and 2 μl/sample were loaded on a 15% SDS-PAGE gel casted according to manufacturer’s protocol (Bio-Rad, Mini-PROTEAN II Electrophoresis Cell). The gels were run at 200 V for 45 min, kept in staining buffer (200 mg Brilliant Blue R 250, 84 ml ethanol, 20 ml glacial acetic acid, and 96 ml water) for half an hour, followed by three washes of at least half an hour each in destaining buffer (75 ml ethanol, 75 ml glacial acetic acid, and 850 ml water) until the bands were clearly visible and no background was present.

### Non-zein protein extraction

 The same flour used above for zein extraction was brought to finer powder in a second step: wrapped in thick aluminum foil and hammered, according to Wu et al., 2012 [28]. This protocol was used for protein extraction and analysis on SDS-PAGE, except for running the gels at 75 V for 3 h instead of 200 V for 45 min.

## Results and Discussions

### Allelic richness, heterozygosity and PIC

The Romanian maize germplasm is a reservoir of genetic diversity. Its potential is best described by the high number of alleles scored among the 90 inbreds that were genotyped with 80 SSR markers. There were a total of 920 alleles, with an average of 11.5/locus. In previous studies the average number of alleles per locus varied from 8.23 [29], to 14.57 [11], to 21.7 [20]. But these numbers have a different meaning when compared to the total number of inbred lines interrogated in the three studies: 154, 1537, and 260, respectively. For a samples size of 90 inbred lines, one would therefore expect an average number of alleles per locus of 4.81 (i.e., 90 x 8.23 / 154) in the first study, 0.85 in the second (90 x 14.57 / 1537), and 7.51 in the third (90 x 21.7 / 260). In the present study a clearer image emerges on the genetic diversity these inbred lines have when comparing these numbers with the 11.5 alleles/locus. The work of Liu et al., 2003 [20] included a set of 260 inbred lines from the U.S., Europe, Canada, South Africa, and Thailand, as well as lines from CIMMYT (International Center for the Improvement of Maize and Wheat) and ITA (Institute of Tropical Agriculture); but no inbred line from SE Europe was included. The same stands true for the work of Yang et al., 2011 [29], where inbred lines originating in the US and China have been used, whereas van Inghelandt et al., 2010 [11] used founder and elite inbred lines from Europe and North-America (but no specific origin is given). 

The observed heterozygosity for the data reported here is extremely low: null for almost half of the markers (36 out of 80), and 0.01 for another 32, with an average of 0.0089. It is of course in accordance with the high values of the inbreeding coefficient (*f*), which has an average of 0.9888 (an expected value, considering that all inbred lines have been self-crossed at least ten times).

 One has to consider the value of PIC (Polymorphism Information Content) for a primer pair before using it for genotyping, and that should be higher than 0.5 in order for the marker to be informative. 73 out the 80 markers used had a value ≥ 0.5, with an average of 0.73. As shown below, at least 70 informative markers are needed to correctly assess population structure and assign an inbred line to its respective cluster/population.

We can therefore conclude that based on the allelic richness, our data are a valuable input towards a better usage of available germplasm in Europe, and the lines described here have a great potential in future breeding efforts. A 2005 study by Reif et al. [30] further substantiates that when analyzing the genetic structure of European flint maize populations. They underline that after World War II, well-adapted Open Pollinated Varieties (OPV) growing here were crossed to high-yielding U.S. dent lines, thus creating the current elite flint lines. However, many OPVs that were not used in the breeding programs may still hold a certain allelic variation that could prove useful for future improvements. 

### The positive/negative traits of local germplasm

**Figure 1:**
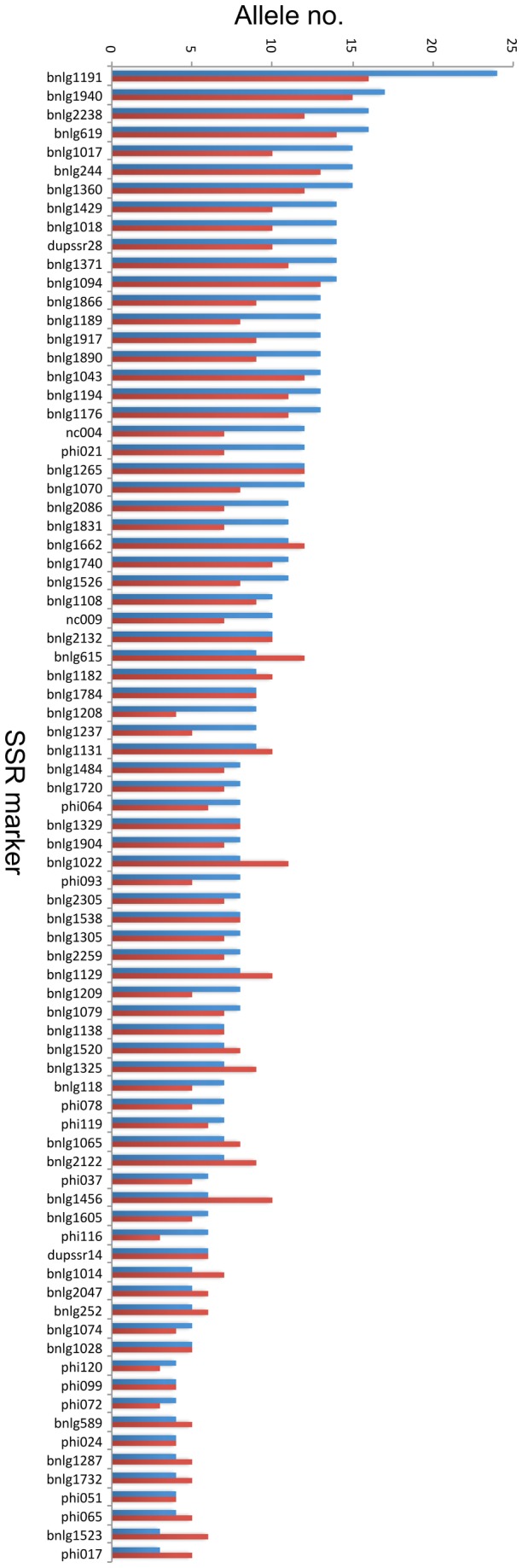
Allelic richness in local germplasm. The number of alleles (y axis) scored at each of the 80 loci (x axis) is compared between a set of 43 local inbred lines (blue bars) and 43 representative inbred lines that are currently being used in breeding programs nationwide (red bars).

In order to stress the importance of local germplasm, the 43 inbred lines used in breeding programs nationwide, were separated from the local germplasm (represented by 47 inbred lines). Four inbred lines from the latter category were excluded to compare the two sets accordingly. The first significant difference was allelic richness, with an average of 9.14 alleles/locus in the case of local germplasm and 7.93 alleles/locus for the other inbred lines. Most of the 80 loci interrogated by SSR markers had more alleles among the local inbred lines (Figure 1), and a statistically higher gene diversity overall (Mann-Whitney test, p = 0.01). Therefore, these lines may indeed represent a genetic reservoir that should be better exploited in the future. 

The main positive trait of local germplasm is the high protein content; statistically higher than for the second category (Mann-Whitney test, p = 0.007). This is important if one seeks to improve the nutritional quality of the grain. If starch content is the trait of interest, then there is a statistical difference in favor of regular inbred lines used for breeding (Mann-Whitney test, p = 0.0004), and that counts as a negative trait for local germplasm. The mean values for fat and fiber are not statistically different between the two classes (data not shown). 

As expected, the local germplasm scores low for morpho-physiological characteristics. Plant height is statistically lower (t-test, p = 0.05), with an average of 155 cm vs. 167 cm, there is a much higher percentage of sterile plants (i.e., without an ear), with an average 28% vs. 12% (Mann-Whitney test, p = 7.084E-05), and 1000 kernels weigh significantly less (203 g vs. 244 g; t-test, p = 0.0003). 

### Population structure

**Figure 2:**
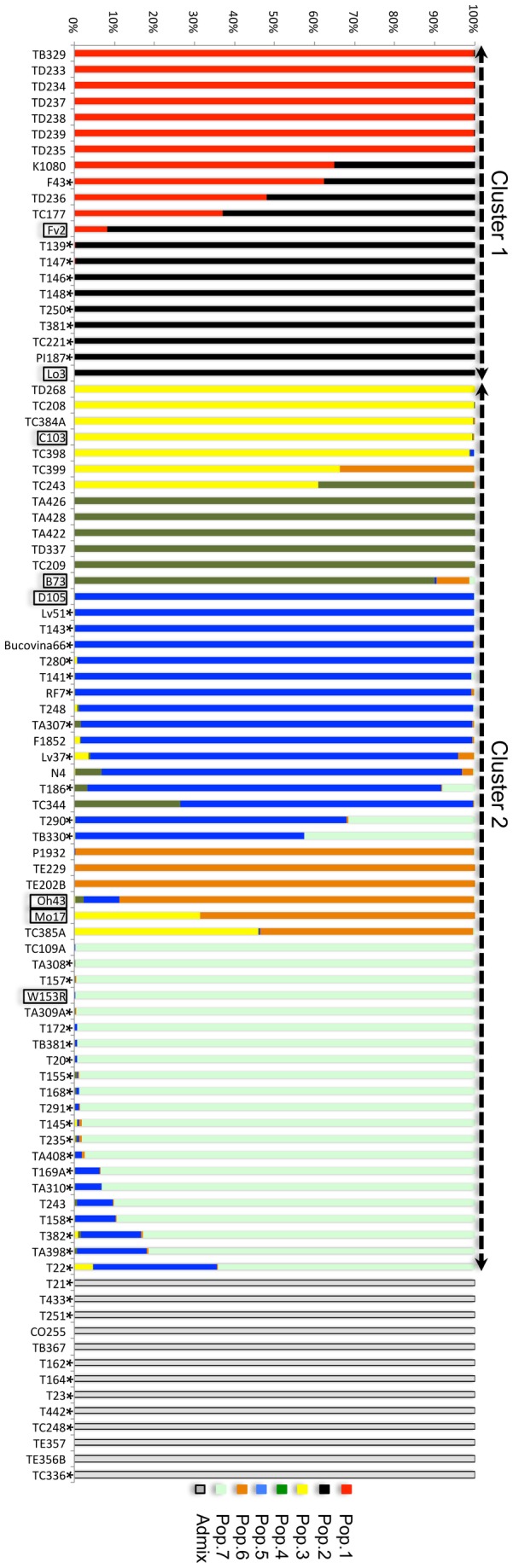
Population structure. The genetic make-up of each of the inbred lines analyzed is represented by 90 vertical lines. They were split in two clusters (dashed arrows on top) using the algorithms implemented in Structure, based on a probability of more than 90%. Those failing the threshold were grouped in an admixed population. The two clusters were further split by the same method in two and five populations, respectively. Each population is color-coded, and segments of different color in the vertical line of an inbred indicate its mixed genetic background (an inbred line is considered as belonging to a particular population if at least 50% of it’s background is shared with the other members). International standards used have a black contour, whereas an asterisk marks local germplasm (i.e., inbred lines with origins in Romanian OPVs).

 The population structure was resolved among the inbred lines analyzed using Structure v.2.3.4. That set the foundation for an easy addition of newly investigated germplasm in the future. As outlined in Material and methods section above, the best K value obtained is two. After splitting the lines accordingly, into two clusters, followed by inference of population structure within the two, there were eight final clusters (referred to as Pop.1 through Pop.8 from now on), including the “admixed” one (i.e., Pop.8) (Figure 2). The first two populations are the result of splitting the first cluster, following the same protocol used in Structure, which resulted in a best K=2. The next five populations are the result of splitting the second cluster according to its inferred best K=5.

**Figure 3:**
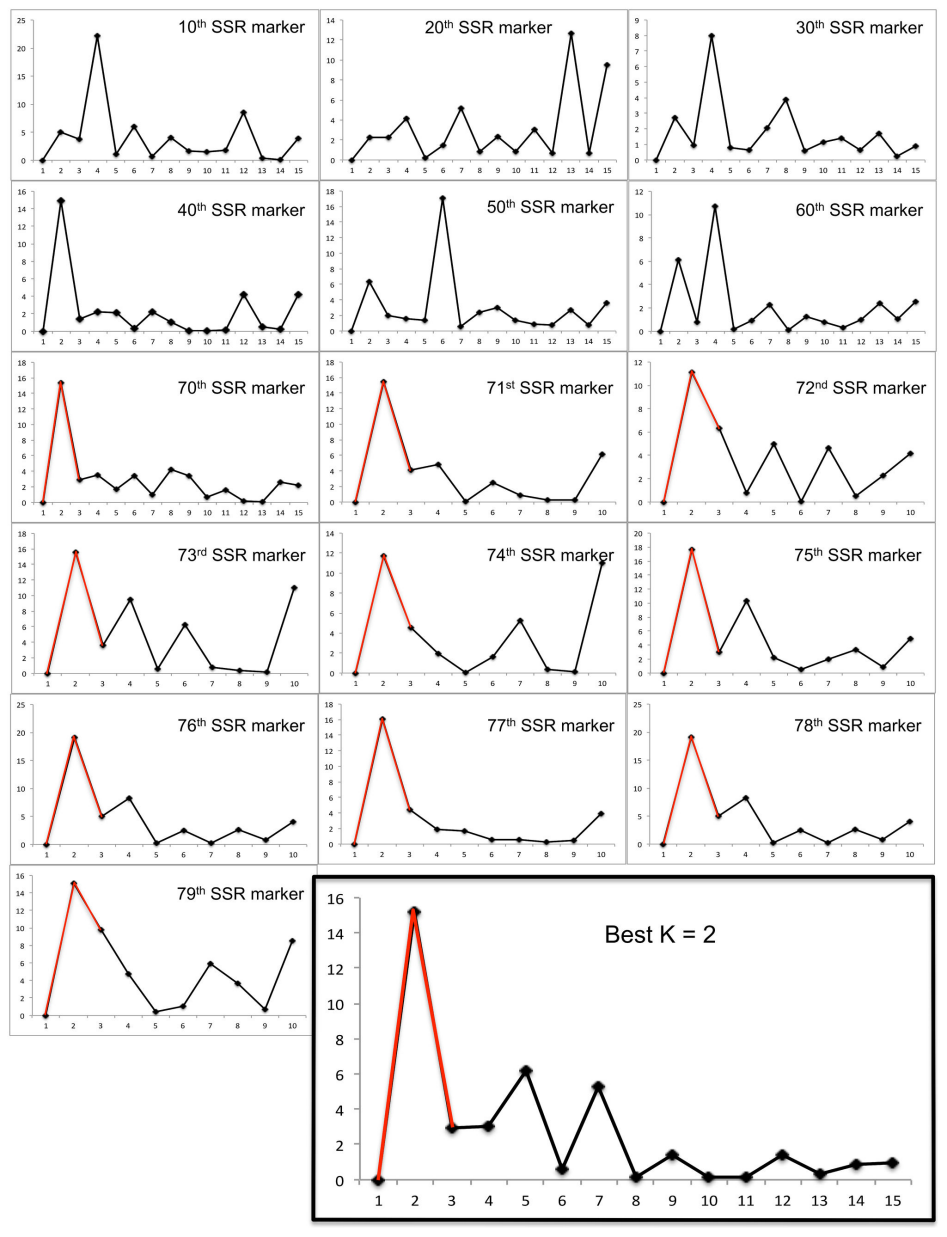
Inference of best K using variable numbers of SSR markers. The best number of clusters (K) to describe population structure is inferred using the output data from Structure and the *ad* *hoc* criterion proposed by [26]. K had variable values when smaller datasets were used (10 to 60 SSR markers). The true K=2 was inferred when using 70 SSR markers, and its value stayed constant on addition of data from one marker at a time (up to the 80 SSRs used). Therefore, 70 informative markers suffice to find the true value of K.

 One must use a minimum number of SSR markers to correctly infer the best K value, otherwise spurious results are generated. For example, best K value was calculated using batches of 10, 20, 30, 40, 50, 60, and 70 SSR markers (Figure 3). It varied between two, four, and six, and even 13 (a highly unlikely value to explain population structure among 90 inbred lines). Nevertheless, this higher value was generated when using 20 SSR markers, which is about double the number of SSRs needed to uniquely fingerprint an inbred line of maize, according to Liu et al., 2003 [20]. In other words, in order to infer a population structure, a much higher number of markers are needed than to simply fingerprint a maize line, and this number is at a minimum 70, as reported here. After reaching this critical number, adding one SSR marker at a time up to 80 did not change the outcome of the population structure, best described by K=2, as shown in Figure 3. We are therefore confident that any inbred line that will be genotyped with these 70 SSR markers (marked with an asterisk in Table S2) in future studies will be easily assigned to one of the already described populations. To the best of our knowledge this is the first estimation of the minimum number of SSRs that are needed to correctly infer population structure among a set of maize inbred lines. Others can therefore benefit from these results, by using just the 70 markers provided and thus avoiding extra-costs and time consuming experiments. All 70 markers have already been optimized for their corresponding annealing temperature, which is available alongside the expected PCR product size in Table S2. 

 Average values for each of the morpho-physiological descriptors used, and for each of the eight populations respectively, are presented in Table 1. Pop.1 scores very high for weight of 1000 kernels, and has very few sterile plants, but scores low for fat content, and behaves poorly to attacks by European corn borer (*Ostrinia nubilalis*). Pop.2 scores highest for protein content, but low for starch and fiber, and they are also generally short plants with a low insertion point for the main ear. Pop.3 doesn’t stand out in terms of agronomically important traits but has the lowest percentage of plants attacked by *O. nubilalis*. Pop.4 scores well for insertion height of the main ear and has a very good kernel efficiency on each ear, with no significant drawbacks. Pop.5 has inbred lines that flower very late and are short. Moreover, it has a high percentage of sterile plants, and has the lowest kernel efficiency. Pop.6 is the richest in starch, but scores lowest for fat content. Its plants reach maturity and flower very late, are significantly taller, and also score highest for weight of 1000 kernels. A negative trait could be the lower insertion point for the main ear. Pop.7 does not score high for any agronomically important trait, except fat (second highest); it has very low values for plant height and the highest number of sterile plants

**Table 1:** Average values for traits of interest across the 8 populations.

***Trait***	**Pop.1**	**Pop.2**	**Pop.3**	**Pop.4**	**Pop.5**	**Pop.6**	**Pop.7**	**Pop.8**
*Pl. hght.(cm)*	165	148.75	167	172.33	152.50	180.17	153.29	176.31
	± 22.52	± 34.40	± 16.34	± 19.45	± 30.78	± 20.7	± 21	± 25.75
*Ear ins. (cm)*	56.22	47.33	49	59	53.63	46.67	51.67	62.62
	± 15.48	± 20.81	± 16.09	± 9.4	± 16.29	± 20.41	± 14.1	± 14.53
*Leaves (no.)*	12	11.83	13	13	13.38	13.67	12.9	13.15
	± 0.82	± 1.52	± 0.93	± 1	± 3.18	± 1.37	± 1.63	± 1.29
*Sterility (%)*	4	20.50	9.71	14.83	27.75	11.83	30.14	16.54
	± 5.79	± 23.86	± 7.85	± 23.58	± 22.56	± 14.55	± 25.71	±16.93
*Ear lgth.(cm)*	13.88	12.90	17.23	15.17	14.71	15.87	14.1	14.6
	± 1.23	± 2.73	± 2.23	± 2.01	± 2.27	± 2.33	± 1.69	± 1.46
*K. eff. (%)*	78.33	77.75	76.86	83.33	74.5	77.17	75.68	80.83
	± 6.51	± 5	± 5.33	± 4.92	± 7.09	± 3.98	± 4.93	± 5.83
*1000 k. (g)*	267.11	169.75	243.71	224.83	236.5	276.17	228.1	185
	± 30.03	± 58.64	± 58.20	± 30.68	± 49.77	± 55.77	± 47.86	± 31.14
*Σ ^o^C to fl.*	665.22	648.33	657.14	669.50	737.38	723.33	629.62	635
	±95.38	± 64.14	± 99.07	± 86.72	± 125.12	± 99.48	± 51.17	± 45.34
*Σ ^o^C to mat.*	1178.06	1107.08	1160.86	1136.83	1082.57	1226.5	1045.33	1056.15
	± 100.51	± 71.76	± 111.34	± 109.08	± 256.14	± 137.17	± 66.11	± 68.47
*O.n att. (%)*	71.22	65.17	60.71	66.83	68.44	65.67	63.05	62.62
	± 7.49	± 20.02	± 20.95	± 16.35	± 17.83	± 11.22	± 13.16	± 28.48
*Protein (%)*	12.93	14.27	12.73	12.53	12.78	12.95	13.04	13.5
	± 0.65	± 1.77	± 1.07	± 1.2	± 0.92	± 0.7	± 1.13	± 1.85
*Fat (%)*	3.6	4.28	4.13	4.02	4.33	3.57	4.34	4.51
	± 0.44	± 1.11	± 0.84	± 0.45	± 0.95	± 0.65	± 0.68	± 0.47
*Starch (%)*	68.48	65.31	68.27	68.55	66.91	69.5	67.35	65.75
	± 1.35	± 3.77	± 1.57	± 1.51	± 2.44	± 1.21	± 2.19	± 3.19
*Fiber (%)*	3.93	3.72	4.16	3.95	3.89	4.03	4.08	4.28
	± 0.72	± 1.95	± 1.12	± 1.13	± 1.42	± 1.43	± 1.33	± 1.16

Averages for 14 traits of interest are compared across the eight populations. As in the case of Table S1, these are: *plant height, insertion height of main ear, number of leaves per plant, percentage of sterility* (i.e., plants not bearing ears), *ear length, kernel efficiency* (percentage of the ear that bears kernels; successful pollination), *weight of 1000 kernels, sum of physiologically active temperatures* (i.e., in the range of 10-30 °C) needed to *flower* and to reach *maturity*, respectively, *percentage of plants attacked by Ostrinia nubilalis, protein, fat, starch, and fiber content.*

 As shown in Figure 2, population structure is shaped around the reference inbred lines included in the analysis: Fv2, Lo3, D105, C103, B73, Oh43, and W153R. The first three are European flints brought from France, Italy, and Germany, widely used after WW II in crosses with American dents [30]; they are represented by the last four lines. The Romanian inbred line TB329 plays a central role in the structure of Pop.1, which is linked to Pop.2 through Fv2. The six lines positioned immediately after TB329 in the figure, plus TD236, form the main body of Pop.1. They are all the result of crosses between TB329 and Fv2. The only inbred line in Pop.1 originating from OPVs of Romania is F43. Conversely, this is not the case for Pop.2, for which all inbred lines clustered around Lo3 are of Romanian origin. Considering that Italian germplasm has started entering Romania in the 19^th^ century [3], it is likely that at least some inbred lines are grouped together with representatives of such origin. Pops.3, 4, and 6 are composed of representative inbred lines that are either Stiff Stalk (SS) – Pop.4 – or Non Stiff Stalk (NSS) – Pops. 3 and 6, which are the main two germplasms used for creating hybrids in the U.S. As expected, Pop.4 is shaped around B73 (the classical SS line), whereas Pops.3 and 6 include C103, Mo17 (the classical SSS line), and Oh43, all three originating in the Lancaster Sure Crop [31]; the first two being very much interrelated (as shown by the big yellow segment of Mo17, which betrays its origin in C103). These three populations are examples of the massive import of North American germplasm into Romania after WW II, containing only inbred lines created afterwards that do not relate in any way with the local germplasm. This is best illustrated by the lack of any such inbred line in these populations. Pops.5 and 7 are mainly composed of such inbred lines, represented by 29 entries out of the total 37. A few of those, share part of their genetic make-up with the sister population, for example. T290 and TB330 have a strong Pop.7 background (represented by the big light-green segments). Conversely, the other way around is also true, for lines such as T22 or TA398 (depicted in their blue segments). However, there is a difference between the two populations: Pop.5 connects Romanian germplasm to central European flints, whose standard is the German D105 line, whereas Pop.7 is shaped around the North American W153R from Minnesota. The majority of inbred lines grouped in the admixed Pop.8 have been originated in the local Romanian germplasm. Since maize has been growing here for centuries, being introduced in the Balkans probably by the Turks [32], it most probably developed into local populations that were later used in creating those inbred lines. Perhaps, this could explain their grouping in an admixed population that cannot be anchored to the international standard germplasm used in the analyses. 

**Figure 4:**
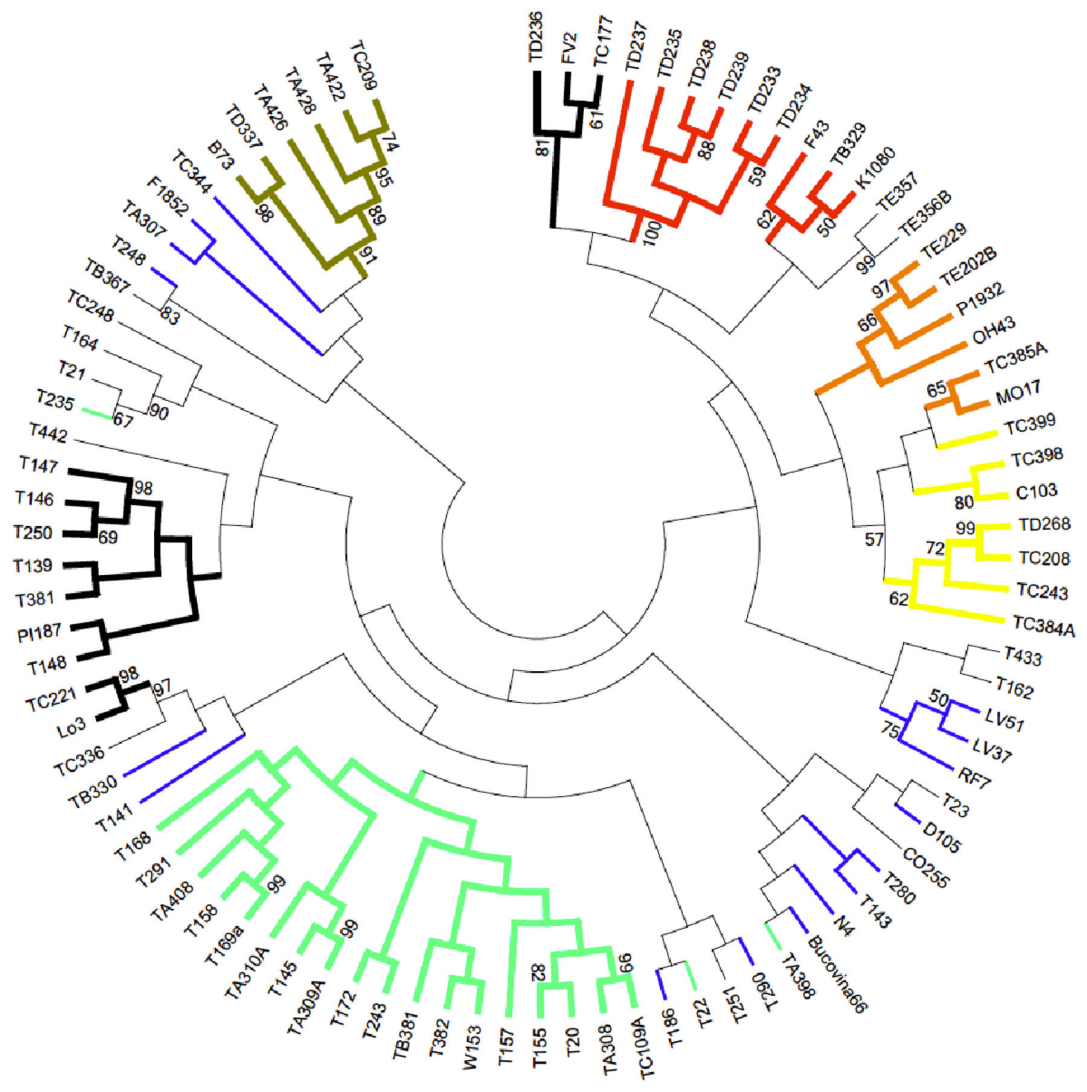
Phylogenetic relationships existing between the 90 inbred lines (NJ tree). The phylogenetic tree reflects population structure inferred by Structure with good fidelity. Color-codes are the same as those used in Figure 2. Bootstrap values higher than 50 are shown on branches.

 There is a very good correlation between the clusters/populations computed by Structure and the consensus Neighbour Joining (NJ) tree, computed in PowerMarker, and visualized in MEGA (Figure 4). Pop.5 doesn’t form a solid cluster of its own, due to the very mixed genetic backgrounds of its members, but it is nonetheless grouped with members of Pop.7. These are two populations that include the majority of local inbred lines used in the present study. The close relationship existing between Pops.3 and 6 is also clearly visible. Pop.4, with its SS lines is very well supported by bootstrap values, but this may also be due to its small number of member lines. High bootstrap values confirm the existing pedigree information for lines TD233, TD234, TD235, TD237, TD238, and TD239 (all coming from the same breeding program). These values also shed new light on the close ties existing between some old local inbred lines and modern ones currently being used in breeding programs, such as TA308 and TC109A. The UPGMA consensus tree has the same topology as the NJ tree (data not shown).

### Correlations between population structure and morpho-physiological, and biochemical characteristics

#### a): Cluster 1 is mainly composed of flint lines

 When kernel type is taken into account, a clear distinction is made between the members of Pop.1 and Pop.2 (flints or semi-flints, except TB329) with Pop.3 and Pop.4 (mostly composed of dent and semi-dent lines, except TA422) (Figure S1). This is a valuable piece of information for breeders, who are seeking for superior hybrids in crosses between members of different heterotic groups. It is worth mentioning that Pop.1 and 2 form together one of the two original clusters inferred using Structure. Therefore, in order to take full advantage of the heterosis phenomenon one should ideally cross inbred lines that are part of this cluster with any of those that are part of the second one. This is based on the reasoning that the further apart two inbred lines are genetically, the more likely it is that the hybrid will show a higher level of heterosis [10]. This is not always the case and depends on the particular trait of interest. For example, Stupar el al., 2008 [33], showed that intra-heterotic group crosses lead to higher level of heterosis when compared to the inter-heterotic ones for the five traits they analyzed: plant final height, days to flowering, weight of 50 seeds, 11-day height and 11-day biomass. In this context, crosses among flint lines of cluster one (i.e., between Pop.1 and Pop.2) may prove more useful.

#### b) Cob color differentiates Pop.1 from Pop.2

A notable difference between the flints of Pop.1 and those of Pop.2 in terms of cob color is visible in Figure S2. The first population has only (dark) red cobs, whereas the latter has only white cobs. Once again, these are the two populations that form cluster one. The cob color is the result of the phlobaphene biosynthesis pathway that is controlled by the R2R3 Myb-like transcription factor, encoded by the *p1* locus in maize [34]. Most importantly, *p1* together with *p2* are two QTLs (Quantitative Trait Locus) for maysin production, a C-glycosyl flavone conferring resistance to corn earworm (*Helicoverpa zea*) [35]. For breeding purposes it is therefore worth investigating the resistance of inbred lines coming from the two populations. In a comparative study, one only uses the germplasm that proves to be more resistant. Most importantly, it could possibly link a visible phenotypic trait to resistance to this agricultural pest.

#### c): Protein, fat, starch, and fiber composition

 There were seven entries (four in Pop.2, and three in Pop.8) that had very high protein content, whereas another 12 had very low levels, when compared to average (Figure S3). The first seven were all inbred lines from the group of 47 originating from local populations of maize (see Material and methods). Among the latter category, only T157, T291, and T169A were inbred lines from local germplasm, with the other nine being part of the contingent of representative inbred lines currently being used in breeding programs. There seems to be a trend, towards increasing starch content which in consequence decreases total protein [36]. This may not have an impact in well-developed countries, but for those where maize is staple food (e.g., countries in Africa and Central America) it can lead to nutritional deficiencies since more than 50% of the human diet is made up of this grain [37]. As shown in Figure S3, Pop.2 has a very good potential in generating a hybrid with high protein content, as we identified four lines scoring high for the trait. This hybrid may be the result of a cross between any of the four lines (i.e., T139, T146, T381, and TC221) and a dent line from Pop.3-7, characterized by an average protein content.

 Two inbred lines, T381 and TC221, scored “high” for protein, fat, and fiber. In consequence, these lines had low levels of starch. We consider them as representatives of local maize germplasm, which harbor great gene diversity and therefore could prove very useful for breeders. Nevertheless, in order to harvest its full potential, one has to take into consideration its disadvantages. For example, T381 is highly susceptible to attacks by the corn borer, and the percentage of sterile plants is over 50%. Such disadvantages must be dealt with through crosses with other inbred lines, preferably from another heterotic group. Hence, generating more viable new hybrids and still scoring high for the three traits mentioned above (protein, fat, and fiber). In this case, parental lines from any of Pops.3 to 7 could prove useful in generating such progeny. Furthermore, TC221 seems to be resistant to attacks and has less than 10% sterile plants, which makes it a perfect candidate as a parental line for future superior hybrids. 

 TC221 illustrates how studies like the present one can significantly reduce cost and labor necessary for field evaluation of a multitude of possible crosses between a set of inbred lines. TC221 helps to point out the existing relationships between them and helps the breeder choose the mating partner from a population of different genetic make-up. 

### Protein patterns confirm the germplasm’s diversity

**Figure 5:**
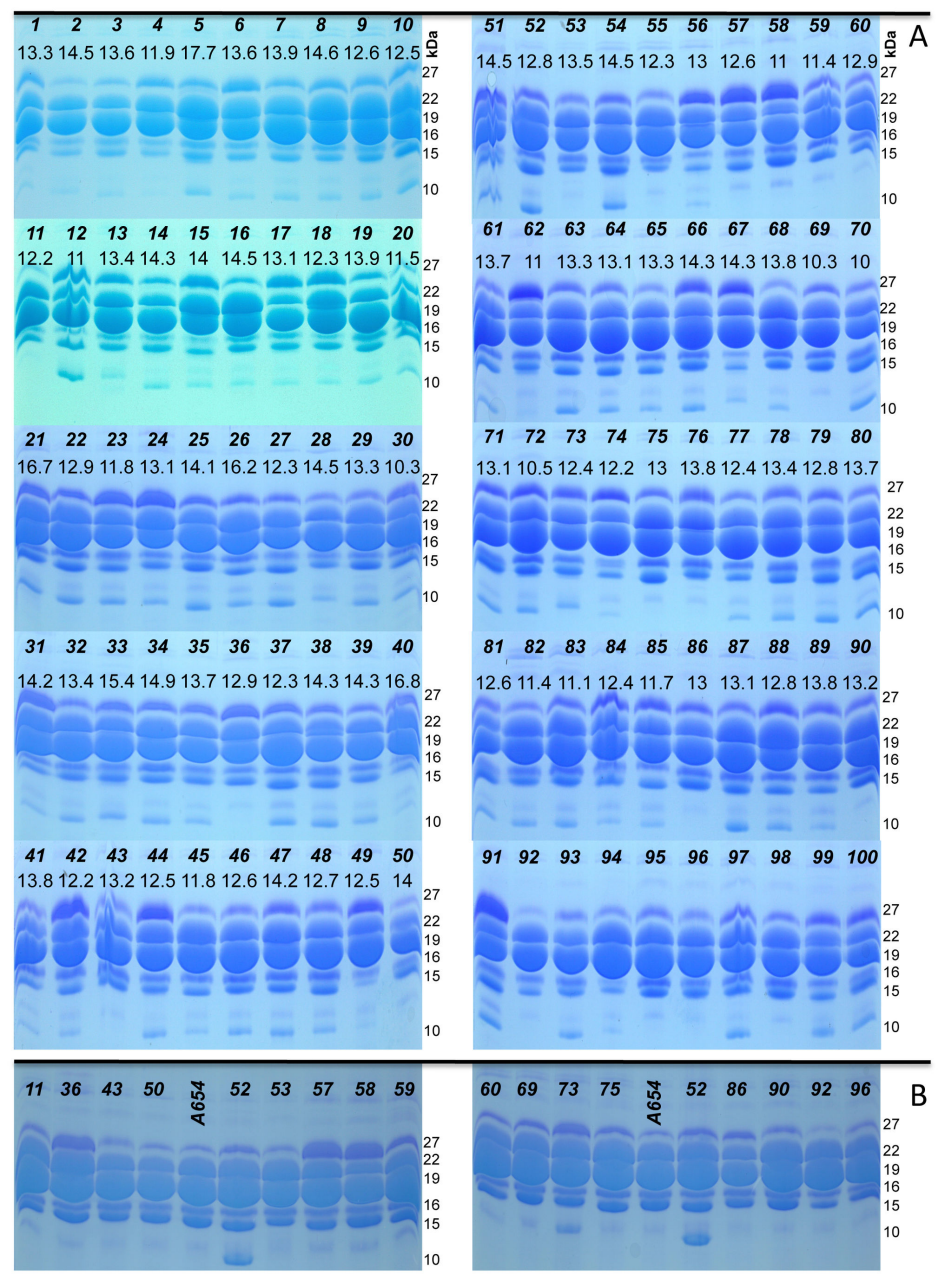
Gel patterns of zein proteins. The zein protein content for 100 inbred lines (numbered 1 to 100) was analyzed by SDS-PAGE (Coomassie Blue staining). The numbers on the top rows of each section match the names of the inbred lines in Table S1. Inbred lines 90-100 are: K1080, A344, TA452, F7, F9, F91, F134, F157, F91a, ICAR54, and Sint1. The values for total protein content (in percentages) are extracted from Table S1 and presented on the bottom rows (except for the newly added ten inbred lines above). Equal amount of protein was loaded in each well and photos were taken using a light box. The characteristic 6-bands pattern (27-, 22-, 19-, 16-, 15-, and 10-kDa) is present for all 100 inbred lines except 22, which are lacking the 10-kDa band (A). 18 of those are compared to inbred line A654 (a natural null mutant for this gene [40]) and to a positive control (inbred #52), from our germplasm, characterized by an intense 10-kDa band (B).

 Among the 100 inbred lines that were used in the analysis (90 that were genotyped, plus an extra ten: K1080, A344, TA452, F7, F9, F91, F134, F157, F91a, ICAR54, and Sint1) the γ-zeins had the highest variability (Figure 5A). They are reported to be the oldest members of the family [38] and might have played a role in maize domestication [39]. Variability extends further in the case of the 10-kDa zeins, where 22 of the samples are completely missing the corresponding band. To confirm its absence, 18 out of the 22 inbred lines were compared to A654, an inbred line described in Wu et al., 2009 [40] as having a null allele for the 10-kDa zein gene. Indeed, the band was absent in all the lines tested against this mutant (Figure 5B). However, the existing variability among zein proteins does not extend to non-zein proteins, all 100 inbred lines having similar patterns (Figure S4).

 The 27-kDa γ-zein has been shown to act as an allergen for early-weaned pigs [41]. As a consequence, the inbred lines that are characterized by high levels of this protein should be avoided in the flour fed to them. Conversely, the 10-kDa zein is very rich in methionine and, if absent, the grain quality is negatively affected. It has been speculated that the high methionine trait, characteristic of exotic maize and teosinte, was lost when this crop was domesticated [42]. However, a present day inbred line, BSSS53, still shows high levels of methionine due to the high expression of the 10-kDa δ-zein [40,43]. Among the 100 inbred lines, not including the 22 that completely lack the 10-kDa band, about half show intermediate methionine levels and another quarter are characterized by a very intense band. Therefore, the last ones have good potential in creating hybrids that are characterized by higher levels of this essential amino acid, thus avoiding the use of transgenics or the addition of synthetic methionine. 

##  Conclusion

 This is the first comprehensive study on the existing SE European maize germplasm in terms of its genetic, morphological, physiological, and biochemical characteristics. It links this germplasm to the international standards used in maize breeding. Due to the allelic richness they hold, the inbred lines presented here are an important addition to the ever-shrinking gene pool, which breeding programs are faced-with nowadays. At the molecular level, local inbred lines developed here constitute a reservoir of genetic diversity. New crosses can be envisioned among their members by unveiling their population structure and defining heterotic groups anchored to international standards. As a result, new hybrids can potentially be generated, which are characterized by high level of heterosis, a phenomenon that translates into higher yield and better seed qualities. 

## Supporting Information

Figure S1 **Many of the flinty lines are clustered in Pops.1 and 2, whereas mostly dent lines form Pops.3 and 4.** The names of inbred lines in Figure 2 were replaced by kernel type, to show the preponderant clustering of flint (F) or semi-flint (s-F) lines in Pops.1 and 2, whereas Pops.3 and 4 are mainly formed by dents (D) or semi-dents (s-D).
(PDF)

Figure S2 **Cob color clearly differentiates Pop.1 from Pop.2.** The names of inbred lines in Figure 2 were replaced by cob color to differentiate the two populations forming cluster 1.
(PDF)

Figure S3 **Vertical comparison of protein, fat, starch, and fiber content in the 90 inbred lines.** Identical to Figure 2, vertical lines represent inbred lines with their respective names on the bottom row. Each inbred line is scored as “Low”, “Average”, or “High” according to its content in the four traits of interest. The average and standard deviation (SD) were calculated among the 90 inbred lines for protein, fat, starch, and fiber. When an inbred line had a value in the interval [average ± one SD], it was scored as “Average”. A value higher than [average + one SD] labels that inbred as “High”. Conversely, a value lower than [average – one SD], translates to a “Low” label.
(PDF)

Figure S4 **Gel patterns of non-zein proteins.** The numbers match the names of the inbred lines in Table S1. Zein and non-zein proteins were migrated in pairs for all 100 inbred lines.
(PDF)

Table S1 **Morphological, physiological, and biochemical descriptors used in characterizing the 90 inbred lines.** All inbred lines (90) in the first column have been characterized by various descriptors, presented in the 23 numbered columns: #1 classifies the inbred lines according to their origin in *Local* (from Romanian local populations), *Int. standard* (8 lines of European or North American origin) used as reference, and *Rep. inbred* (representative inbred lines of Romania, currently being used in breeding programs nationwide); #2 to #5 present the plant morphology in terms of height, insertion height of the main ear, number of leaves per plant and percentage of plant without an ear (i.e., sterile); #6 to #10 describe ear morphology; #11 presents the sum of physiologically active temperatures (that are in the range of 10 - 30 °C) needed to flower, whereas #12 is the sum needed to reach maturity; #13 is percentage of plants that were attacked by *Ostrinia nubilalis*; #14 to #17 represent percentages of each important component of maize kernel (i.e., proteins, fat, starch, fiber); #18 to #23 are the scores of each inbred when the gel image was analyzed (since the 27-kDa band was the most variable, its intensity was scored from 1 to 4, the last being the most intense. The other five bands were scored from 1 to 3; values of 0 mean that the band was missing in that inbred lines).
(XLS)

Table S2 **Primers used.** The chromosomal position, repeat type, forward and reverse sequences for each of the 80 SSR primer pairs used is shown in the first columns. The annealing temperature was optimized for specific amplification (the PCR machines were Eppendorf ep*gradient* S) in each case. The observed size is a range of sizes (i.e., alleles) for each primer pair, as scored by GeneMapper v.4.0 data analysis software on the 90 inbred lines.
(XLSX)

Table S3 **Raw table, in the required PowerMarker v.3.25 format.** The synthetic table contains all data generated for the 90 inbred lines and can be easily added to other such files, generated in other laboratories, for comparative analyses, for filling-up missing data or for enlarging their dataset. It contains the 90 inbred lines in the first column, followed by 160 columns: two for each SSR marker (ideally, both columns of a marker should be identical, since the inbred lines are expected to be homozygous at all loci, but is some very few cases the locus is heterozygous; as shown in the main text, heterozygosity was very low for all 90 inbred lines though). Missing data is marked by the “?” sign.
(TXT)

Table S4 **Raw table, in the required Structure v.2.3.4 format.** The Structure format is very similar to the PowerMarker format but the two columns for each marker become two rows for each of the 90 inbred lines. All other information from the legend of Table S3 stays the same, with the sole exception that missing data is marked by “-9” instead of “?”.
(XLS)
