# Correction: Romanian Maize (*Zea mays*) Inbred Lines as a Source of Genetic Diversity in SE Europe, and Their Potential in Future Breeding Efforts

**DOI:** 10.1371/annotation/85f0b730-b723-4d59-a653-d48b974c366c

**Published:** 2014-01-14

**Authors:** Dana Șuteu, Ioan Băcilă, Voichița Haș, Ioan Haș, Mihai Miclăuș

The Editor of this article was mistakenly omitted from the final document. The publisher apologizes for the error. The Editor is:

Jinfa Zhang, New Mexico State University, USA.

