# Supplementary figures and images for: Romanian Maize (Zea mays) Inbred Lines as a Source of Genetic Diversity in SE Europe, and Their Potential in Future Breeding Efforts
Source: PLoS One. 2013 Dec 31;8(12):e85501. doi: 10.1371/journal.pone.0085501 (PMC3877385; doi:10.1371/journal.pone.0085501)

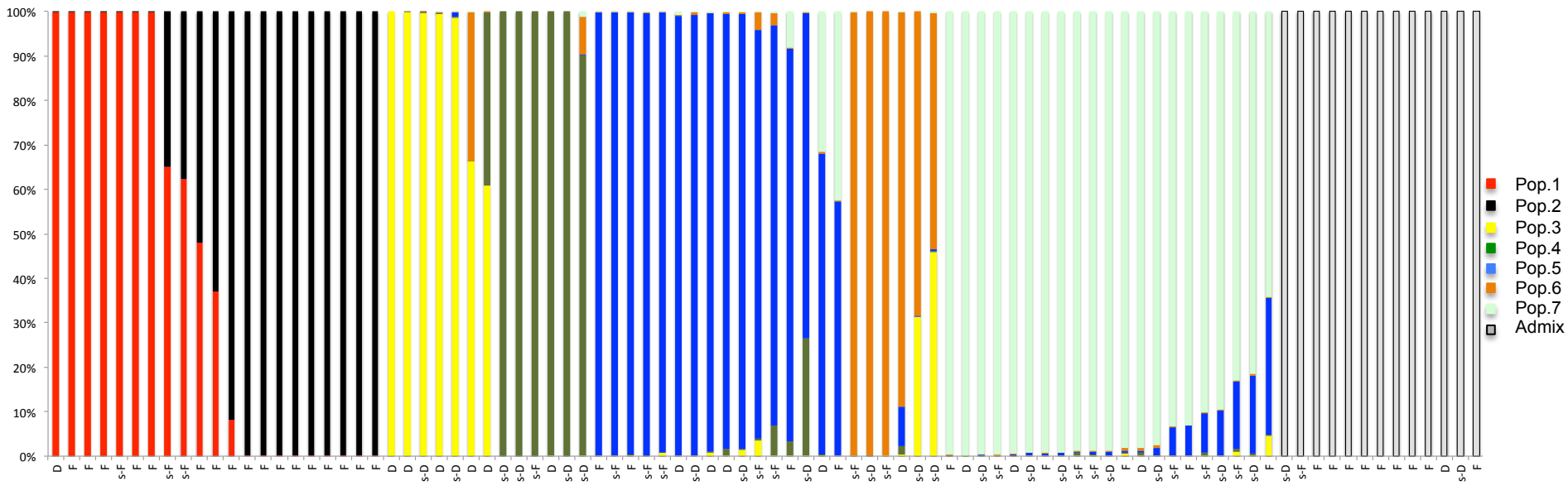

Supplement: Figure S1 — Many of the flinty lines are clustered in Pops.1 and 2, whereas mostly dent lines form Pops.3 and 4. The names of inbred lines in Figure 2 were replaced by kernel type, to show the preponderant clustering of flint (F) or semi-flint (s-F) lines in Pops.1 and 2, whereas Pops.3 and 4 are mainly formed by dents (D) or semi-dents (s-D). (PDF) [file pone.0085501.s001.pdf]

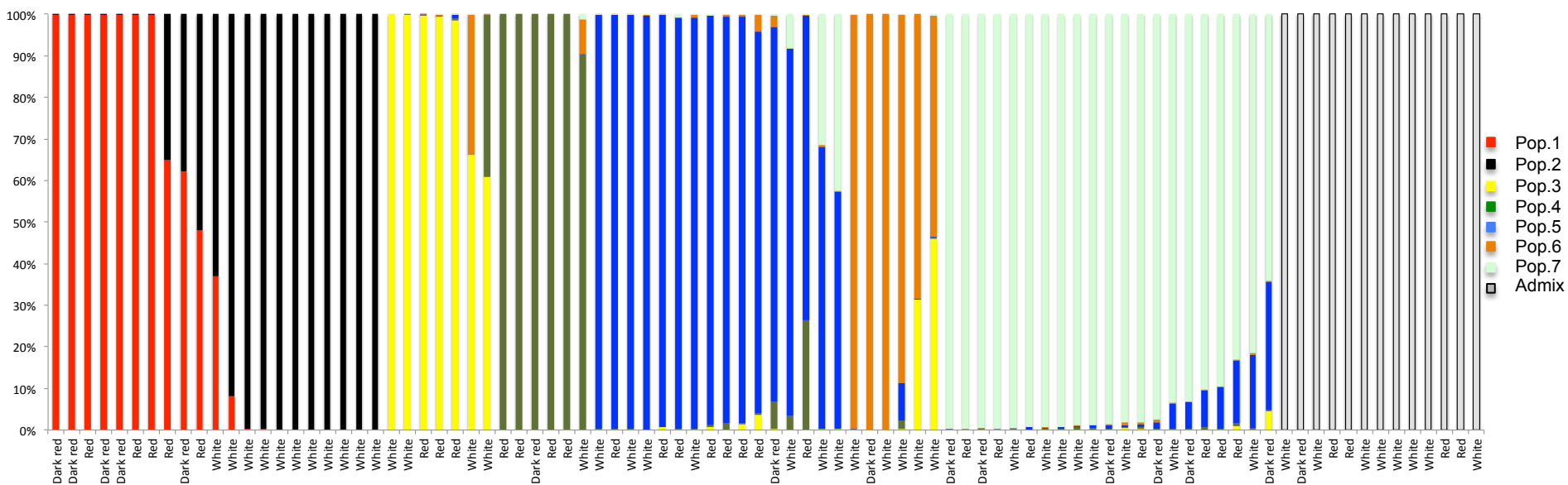

Supplement: Figure S2 — Cob color clearly differentiates Pop.1 from Pop.2. The names of inbred lines in Figure 2 were replaced by cob color to differentiate the two populations forming cluster 1. (PDF) [file pone.0085501.s002.pdf]

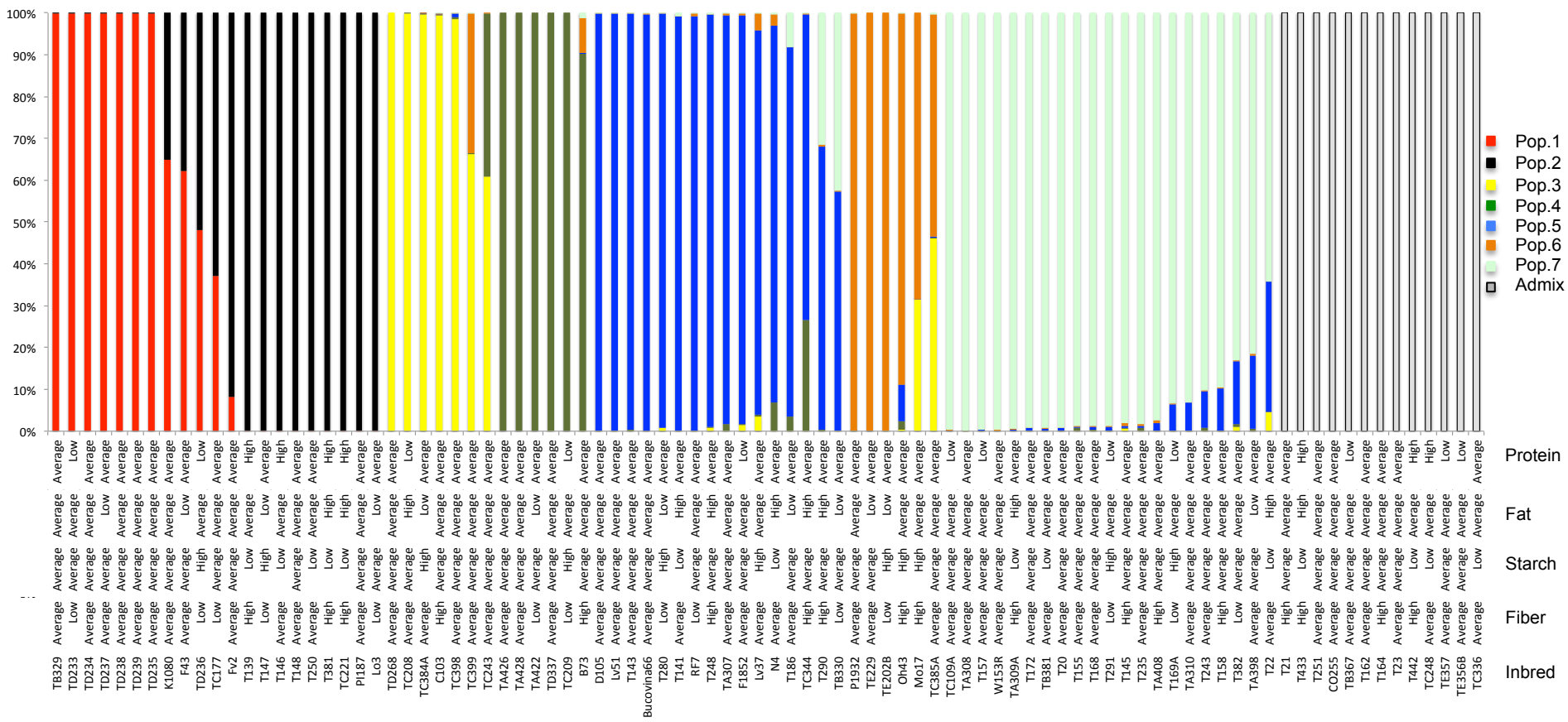

Supplement: Figure S3 — Vertical comparison of protein, fat, starch, and fiber content in the 90 inbred lines. Identical to Figure 2, vertical lines represent inbred lines with their respective names on the bottom row. Each inbred line is scored as “Low”, “Average”, or “High” according to its content in the four traits of interest. The average and standard deviation (SD) were calculated among the 90 inbred lines for protein, fat, starch, and fiber. When an inbred line had a value in the interval [average ± one SD], it was scored as “Average”. A value higher than [average + one SD] labels that inbred as “High”. Conversely, a value lower than [average – one SD], translates to a “Low” label. (PDF) [file pone.0085501.s003.pdf]

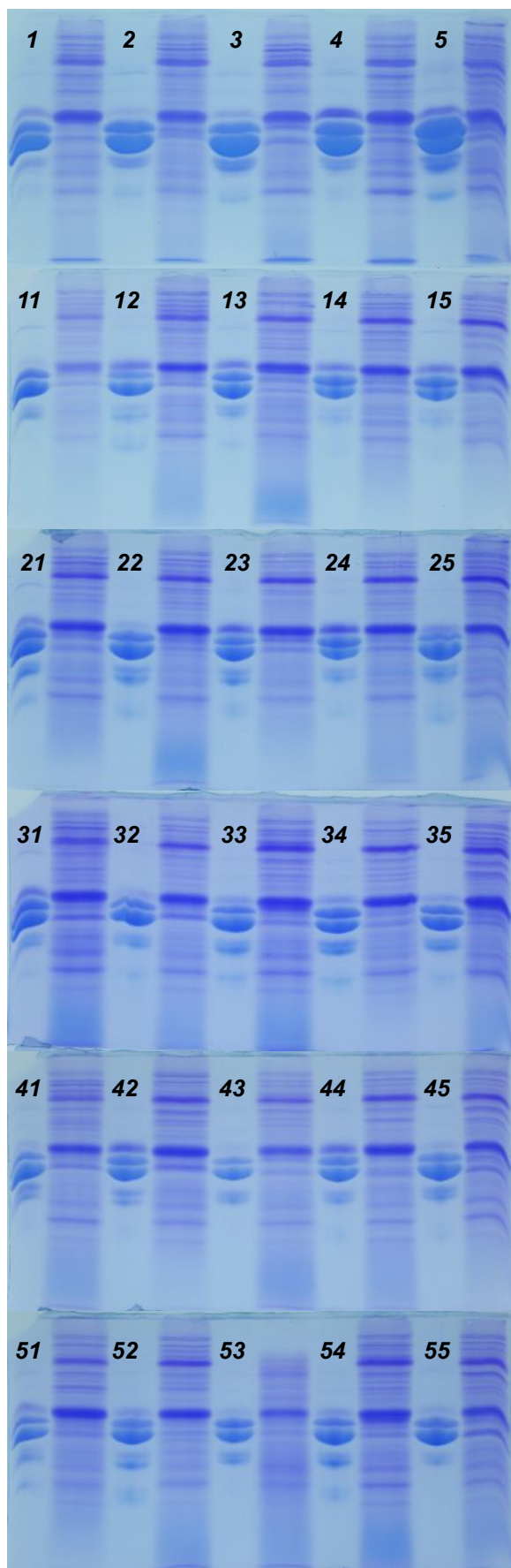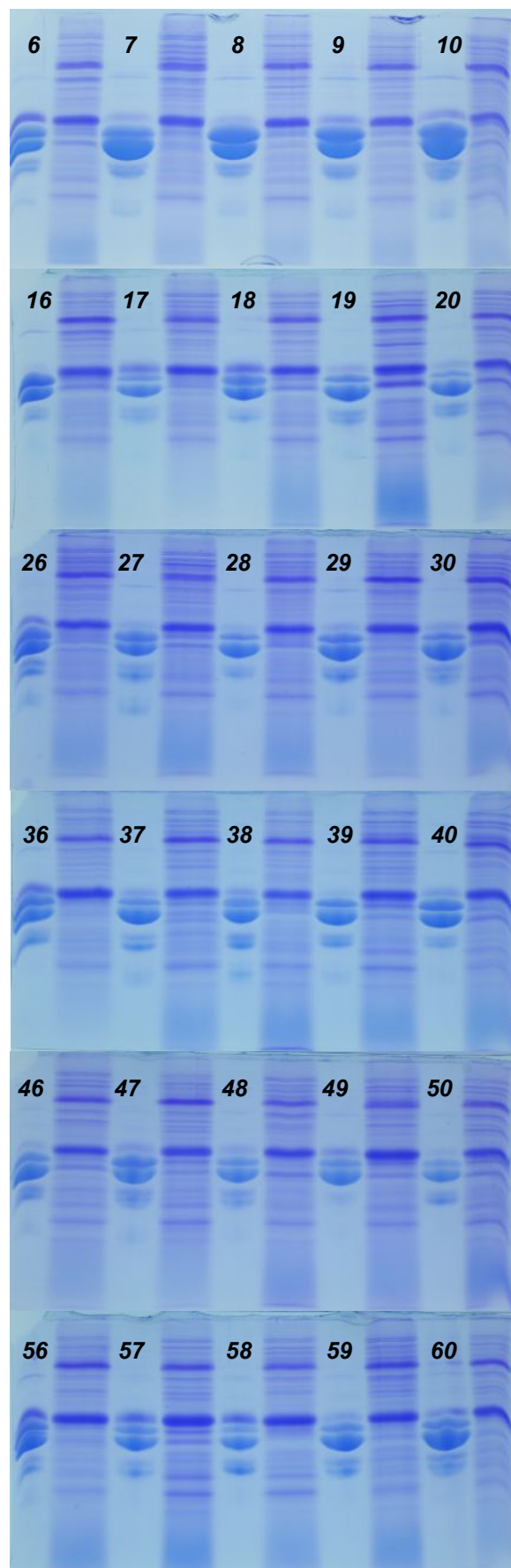

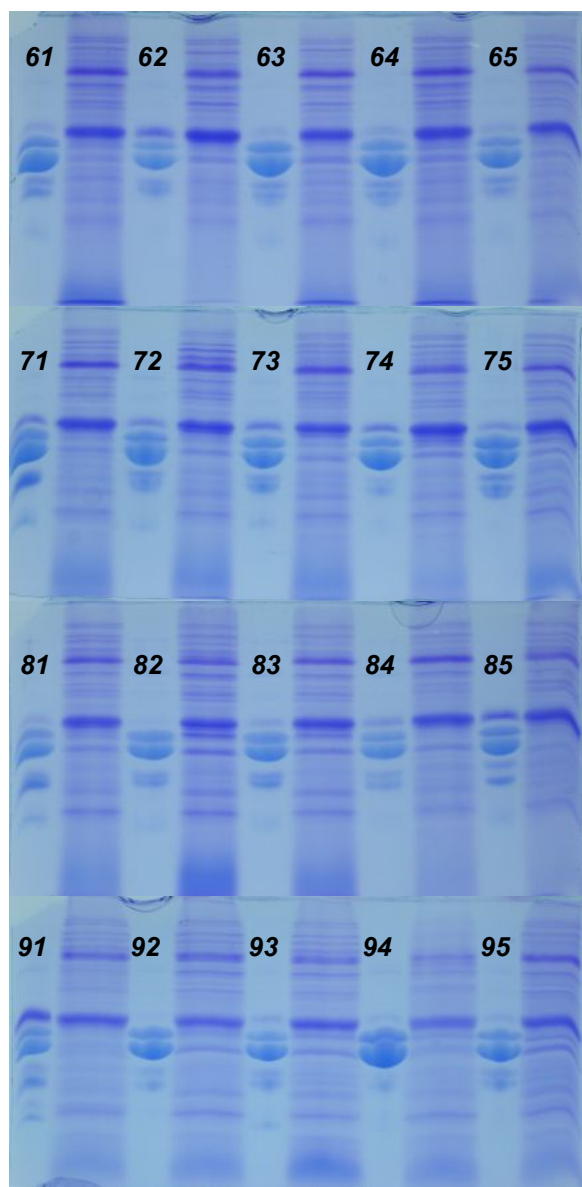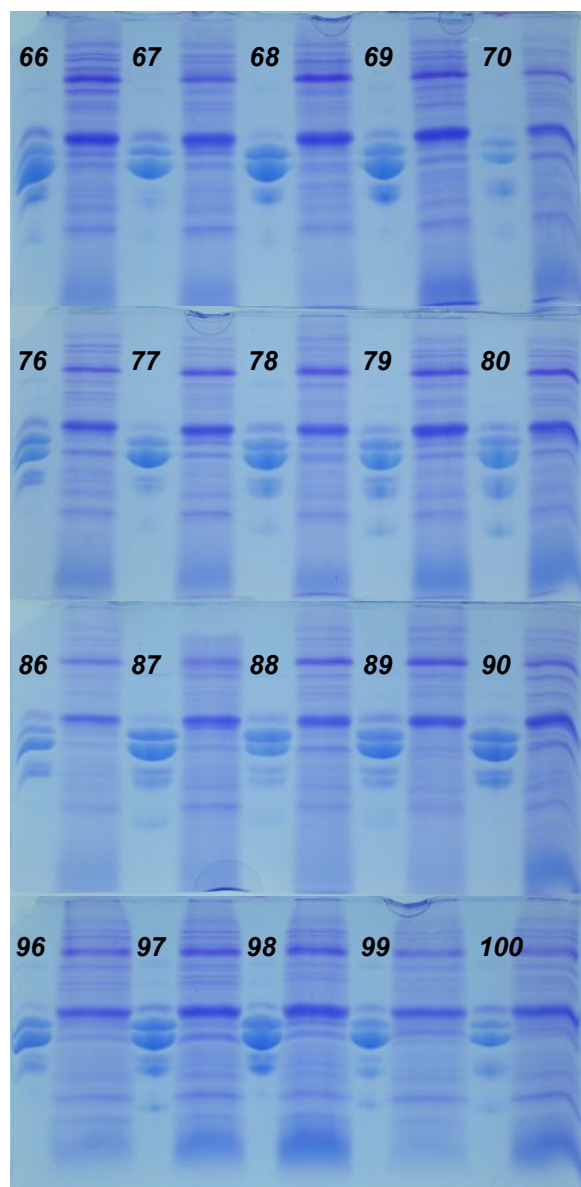

Supplement: Figure S4 — Gel patterns of non-zein proteins. The numbers match the names of the inbred lines in Table S1. Zein and non-zein proteins were migrated in pairs for all 100 inbred lines. (PDF) [file pone.0085501.s004.pdf]
